# Association of Ferredoxin:NADP^+^ oxidoreductase with the photosynthetic apparatus modulates electron transfer in *Chlamydomonas reinhardtii*

**DOI:** 10.1007/s11120-017-0408-5

**Published:** 2017-06-07

**Authors:** Laura Mosebach, Claudia Heilmann, Risa Mutoh, Philipp Gäbelein, Janina Steinbeck, Thomas Happe, Takahisa Ikegami, Guy Hanke, Genji Kurisu, Michael Hippler

**Affiliations:** 1 Institute of Plant Biology and Biotechnology, University of Münster, Schlossplatz 8, 48143 Münster, Germany; 2 Institute for Protein Research, Osaka University, 3-2 Yamadaoka, Suita-shi, Osaka, 565-0871 Japan; 3 Department of Plant Biochemistry, Ruhr-University Bochum, Universitätsstrasse 150, 44801 Bochum, Germany; 4 Structural Epigenetics Laboratory, Graduate School of Medical Life Science, Yokohama City University, 1-7-29 Suehiro-cho, Tsurumi-ku, Yokohama, 230-0045 Japan; 5 School of Biological and Chemical Sciences, Queen Mary University of London, Mile End Road, London, E1 4NS UK

**Keywords:** *Chlamydomonas reinhardtii*, Electron transport regulation, Ferredoxin:NADP^+^ oxidoreductase, Ferredoxin, Proton Gradient Regulation 5, PGR5-like photosynthetic phenotype 1

## Abstract

Ferredoxins (FDX) and the FDX:NADP^+^ oxidoreductase (FNR) represent a key junction of electron transport downstream of photosystem I (PSI). Dynamic recruitment of FNR to the thylakoid membrane has been considered as a potential mechanism to define the fate of photosynthetically derived electrons. In this study, we investigated the functional importance of the association of FNR with the photosynthetic apparatus in *Chlamydomonas reinhardtii*. In vitro assays based on NADP^+^ photoreduction measurements as well as NMR chemical shift perturbation analyses showed that FNR preferentially interacts with FDX1 compared to FDX2. Notably, binding of FNR to a PSI supercomplex further enhanced this preference for FDX1 over FDX2, suggesting that FNR is potentially capable of channelling electrons towards distinct routes. NADP^+^ photoreduction assays and immunoblotting revealed that the association of FNR with the thylakoid membrane including the PSI supercomplex is impaired in the absence of Proton Gradient Regulation 5 (PGR5) and/or Proton Gradient Regulation 5-Like photosynthetic phenotype 1 (PGRL1), implying that both proteins, directly or indirectly, contribute to the recruitment of FNR to the thylakoid membrane. As assessed via in vivo absorption spectroscopy and immunoblotting, PSI was the primary target of photodamage in response to high-light stress in the absence of PGR5 and/or PGRL1. Anoxia preserved the activity of PSI, pointing to enhanced electron donation to O_2_ as the source of the observed PSI inactivation and degradation. These findings establish another perspective on PGR5/PGRL1 knockout-related phenotypes and potentially interconnect FNR with the regulation of photosynthetic electron transport and PSI photoprotection in *C. reinhardtii*.

## Introduction

The FDX network represents a major hub of chloroplast metabolism. Reduced FDX is considered as a site of fine-tuned electron distribution towards multiple diverging pathways (Hanke and Mulo 2013; Goss and Hanke 2014; Hase et al. 2006). As a [2Fe2S] electron carrier, FDX transfers electrons from PSI to the FDX:NADP^+^ oxidoreductase (FNR). In addition to its primary function in photosynthetic electron transport, FDX contributes to sulphur and nitrogen assimilation via electron donation to the sulphite and the nitrite reductase. Furthermore, FDX donates electrons to the glutamate synthase and the fatty acid desaturase, thereby promoting amino acid and lipid biosynthesis. Moreover, FDX relates photosynthetic electron transport to the thioredoxin (TRX) system of the chloroplast via the FDX–TRX reductase. FDX metabolism in *Chlamydomonas reinhardtii* features two additional special aspects: FDX presumably accepts electrons from the Pyruvate:FDX oxidoreductase (PFR1), providing a link to starch metabolism (Noth et al. 2013); and it donates electrons to the [FeFe] hydrogenase (HydA), mediating H_2_ photoproduction (Hemschemeier and Happe 2011). In cyanobacteria, algae and vascular plants, specific FDX isoforms channel electrons towards distinct metabolic pathways (Razquin et al. 1995; Kimata-Ariga et al. 2000; Hanke and Hase 2008; Terauchi et al. 2009). *C. reinhardtii* features at least six plant-type FDX isoforms (Winkler et al. 2010). The major isoform, leaf-type FDX1, constitutes 99% of the *FDX* transcript pool in regular growth conditions and is likely engaged in most of these processes. FDX5, being induced in anoxia, has been reported to be critical for the chloroplast redox homeostasis in the dark (Jacobs et al. 2009; Yang et al. 2015). Root-type FDX2, being induced in anoxia and during growth on nitrate, is the only other FDX capable of efficiently donating electrons to FNR besides FDX1 (Terauchi et al. 2009; Peden et al. 2013).

Another candidate defining the fate of electrons derived from photosynthetic electron transport is FNR. The flavoenzyme is present in the chloroplast stroma both in a soluble and a thylakoid membrane-bound state. Dynamic association with and dissociation from distinct membrane complexes is considered to contribute to the partitioning of photosynthetic electron transport between linear electron flow (LEF) and cyclic electron flow (CEF) (Joliot and Johnson 2011). According to this FNR model, the association of FNR with PSI (or soluble FNR in the stroma) may promote LEF, while CEF might operate preferentially following the association of FNR with Cyt b_6_f (Clark et al. 1984; Zhang et al. 2001). The precise molecular mechanism of the major CEF pathway in *C. reinhardtii* is still elusive. Although in vitro experiments on vascular plants indicate that PGRL1 may be the catalyst (Hertle et al. 2013), there is no definite evidence in *C. reinhardtii* as to which component supplies the required FDX:quinone oxidoreductase (FQR) activity in vivo. In this context, it has been proposed that plastoquinone (PQ) might be reduced at the Qi site according to a modified Q-cycle process involving concerted electron transfer from FDX or FNR, potentially via heme c_i_, and from heme b_h_ to PQ (Joliot and Joliot 2006; Johnson 2011; Hasan et al. 2013).

In contrast to vascular plants, *C. reinhardtii* possesses only a single FNR, which is evolutionarily closer to vascular plant root-type than leaf-type FNR isoforms (Appendix Fig. 6; Goss and Hanke 2014). This may be since the enzyme has to mediate both FDX-dependent NADP^+^ photoreduction and NADPH-dependent FDX reduction in heterotrophic conditions. In *A. thaliana*, FNR is anchored to the thylakoid membrane via Tic26, TROL and LIR1 (Benz et al. 2009; Juric et al. 2009; Yang et al. 2016). However, in the unicellular alga no orthologues of these tethering proteins have been identified yet. In *C. reinhardtii*, FNR has been reported previously to associate with the PSI–LHCI–LHCII supercomplex (Takahashi et al. 2014; Bergner et al. 2015) as well as with the potential CEF supercomplex comprising Cyt b_6_f, PSI, LHCI, LHCII, PGRL1, PETO, ANR1 and CAS (Iwai et al. 2010; Terashima et al. 2012; Takahashi et al. 2013). In vascular plants, a PSI–Cyt b_6_f multiprotein complex, which however lacks FNR and PGRL1, has been identified in a recent electron microscopy-based study (Yadav et al. 2017). In *C. reinhardtii*, the formation of these multiprotein complexes is induced in response to alterations in the chloroplast redox poise such as shifts to anoxia or high light (Takahashi et al. 2013; Bergner et al. 2015), i.e. conditions where CEF is induced to alleviate stromal redox pressure (Alric 2014). Knockdown of PETO, ANR1 or CAS results in impaired CEF induction upon a shift to anoxia (Terashima et al. 2012; Takahashi et al. 2016). Likewise, the absence of PGRL1 has been attributed with a defect in FQR-dependent CEF efficiency, as has the lack of PGR5 (Munekage et al. 2002; DalCorso et al. 2008). In general, PGR5 and PGRL1 knockout mutants in *C. reinhardtii* and *A. thaliana* feature multifaceted phenotypes, most importantly including deficient qE-dependent NPQ induction (Munekage et al. 2002; DalCorso et al. 2008; Tolleter et al. 2011; Johnson et al. 2014) and severe PSI photodamage in response to high-light stress (Munekage et al. 2002; Suorsa et al. 2012; Johnson et al. 2014; Kukuczka et al. 2014). Despite these distinct phenotypes pointing to perturbation of the transmembrane proton gradient and deregulation of photosynthetic electron transport, the precise molecular roles of PGR5 and PGRL1 in *C. reinhardtii* remain elusive. Besides regulating the activity of PSII via induction of qE-dependent NPQ, ΔpH controls the rate-limiting step of LEF, i.e. the oxidation of PQH_2_ at the Q_o_ site of Cyt b_6_f (Eberhard et al. 2008; Rott et al. 2011). As the induction of qE-dependent NPQ is delayed in *C. reinhardtii*, shifting the rate-limiting step of photosynthetic electron transfer from the PSI acceptor side to Cyt b_6_f is critical, in particular for short-term acclimation to high-light stress (Chaux et al. 2015).

In this work, we further explored how FNR might differentially modulate electron flow downstream of PSI in combination with PGR5 and PGRL1. For this purpose, we studied the interaction of FNR with its substrates FDX1 and FDX2, as well as how this interaction is altered upon binding of FNR to PSI. In addition, we investigated the activity and localization of FNR as well as the high-light response of mutants impaired in photosynthesis regulation (*pgr5, pgrl1* and *pgr5pgrl1*), attempting to bridge the gap between potential molecular mechanisms and physiological impacts.

## Materials and methods

### Strains and growth conditions

The experiments presented here were performed with the *C. reinhardtii* wild-type strain cc124 (137c, nit2-, mt-), a PGR5 knockout strain (*pgr5*) (Johnson et al. 2014), a PGRL1 knockout strain (Tolleter et al. 2011) back-crossed to the cc124 background (Kukuczka et al. 2014) and a PGRL1/PGR5 double mutant (*pgrl1pgr5*) derived from a cross of the respective single mutants (Steinbeck et al. 2015). All strains were maintained on TAP medium (Harris 1989), solidified with 1.5% w/v agar at 25 °C in the presence of ~50 μE m^−2^ s^−1^ photosynthetically active, continuous illumination. For experiments, strains were cultured in TAP medium on a rotary shaker (120 rpm) at 25 °C in the presence of ~20 μE m^−2^ s^−1^ photosynthetically active, continuous illumination. To assess the physiological response to high-light stress (~200 μE m^−2^ s^−1^), cells were set to a chlorophyll concentration of 4 μg mL^−1^ and shifted to photoautotrophic conditions (Fig. 4a) or bubbled with argon to eliminate O_2_ in heterotrophic conditions (Fig. 4b).

### Thylakoid isolation, solubilisation and SDG ultracentrifugation

Thylakoids were isolated as previously described in (Terashima et al. 2012). Prior to the isolation procedure, cultures were bubbled with argon for 4 h producing anaerobic conditions to induce the formation of the putative CEF supercomplex which recruits FNR to the membrane. Cell disruption was performed by means of a Bio*Nebulizer* at ~1.5 bar N_2_ pressure. Isolated thylakoids (0.8 mg chl mL^−1^) were solubilized in the presence of 0.9% β-DDM (700 µL total volume) for 20 min. Separation of solubilised thylakoids to isolate photosystem particles by 1.3–0.4 M sucrose density gradient (SDG) ultracentrifugation was performed as described previously (Takahashi et al. 2006; Terashima et al. 2012).

### SDS PAGE, immunoblot and ECL detection

Samples were adjusted to equal chlorophyll (whole cells and thylakoids) or equal volume (SDG fractions), supplemented with SDS PAGE loading buffer and incubated at 65 °C for 10 min. Proteins were separated by 13% (w/v) SDS PAGE, blotted onto nitrocellulose membranes and detected by specific primary antibodies: AtpB (Agrisera), PsaD (Naumann et al. 2005) and FNR (kind gift of Y. Takahashi, Okayama University). Secondary antibodies were anti-rabbit (Invitrogen).

### Isolation of FDX1, FDX2, PC and FNR for light-dependent NADPH production assays

Recombinant *C. reinhardtii* FDX1, FDX2 and plastocyanin (PC) were expressed in *Escherichia coli*. FDX1 and FDX2 were purified by Strep-tag affinity chromatography, PC was purified via anion exchange chromatography and gel filtration as described previously (Kuhlgert et al. 2012). *C. reinhardtii* FNR was purified as described previously for *Zea mays* FNR (Onda et al. 2000).

### Light-dependent NADPH production assays

Light-dependent NADPH production mediated by isolated PSI and PSI–FNR complexes as well as isolated thylakoid membranes was assessed as described previously (Finazzi et al. 2005). Samples with isolated PSI particles (5 µg chl mL^−1^) contained 20 mM Tricine–KOH (pH 7.4), 3 mM MgCl_2_, 2 mM ascorbate, 60 µM DCPIP, 40 µM PC, 4 µM FDX1/2, 0.5 mM NADP^+^ and 0.5 µM FNR as specified. Samples with isolated thylakoids (66 μg chl mL^−1^) contained 50 mM HEPES–KOH (pH 7.5), 10 mM MgCl_2_, 0.1 μM nigericin, 0.05% β-DDM, 2 mM ascorbate, 60 μM DCPIP, 5 μM PC, 10 μM FDX1, 0.5 mM NADP^+^ and 0.5 µM FNR as specified. NADP^+^ photoreduction assays were performed with both commercially available *Spinacia oleracea* FNR (Sigma-Aldrich) and purified *C. reinhardtii* FNR. While *C. reinhardtii* FNR mediated slightly higher NADPH production rates, the relative rates obtained with FDX1 versus FDX2 were identical. Absorption at 340 nm (and at 390 nm for correction) was measured prior to and after 15 and 30 s (PSI particles) and 20 and 40 s (thylakoids) of saturating illumination with a white halogen lamp (PSI particles) or a tungsten lamp with 610 nm long-pass cut-off filter (thylakoids).

### Isolation of non-labelled and [^15^N]-labelled FDX1, FDX2 and FNR for NMR measurements

Recombinant *C. reinhardtii* FDX1, FDX2 and FNR were expressed in *E. coli* as described previously (Mutoh et al. 2015).

### NMR measurements

Two-dimensional (2D) ^1^H–^15^N heteronuclear single-quantum coherence (HSQC) spectra of [^15^N]-FDXs were recorded on a Bruker Avance III NMR spectrometer with a ^1^H resonance frequency of 950 MHz with a TCI cryoprobe™, as described previously (Mutoh et al. 2015). Mixtures of FDXs and FNR in 25 mM MES–NaOH (pH 6.5) containing 50 mM NaCl, 0.04% β-DDM and 10% D_2_O for the NMR lock were analysed at 298 K. Weighted averages (Δ*δ*_ave_) of ^1^H (Δ*δ*_1HN_) and ^15^N (Δ*δ*_15N_) chemical shift changes were calculated according to the following formula: Δ*δ*_ave_ = [(Δ*δ*_1HN_)^2^ + (0.04Δ*δ*_15N_)^2^]^1/2^, where the parameter 0.04 was derived from the ratio between the ^1^H and ^15^N chemical shift distributions.

### Competition experiments

[^15^N]-FDX1 and FNR were mixed at a molar ratio of 1:3 (50:150 µM) in the absence or presence of non-labelled 150 µM FDX2. The same procedure was followed with [^15^N]-FDX2. The weighted average of chemical shift changes (Δ*δ*_ave_) was calculated for each peak. To compare Δ*δ*_ave_ of [^15^N]-FDX1 + FNR in the absence of FDX2 (Δ*δ*_without FDX2_) with that of [^15^N]-FDX1 + FDX2 + FNR in the presence of FDX2 (Δ*δ*_with FDX2_), the ratio of Δ*δ*_with FDX2_ to Δ*δ*_without FDX2_ was calculated. Under a fast exchange regime on the NMR time scale, Δ*δ*_with FDX2_/Δ*δ*_without FDX2_ would become smaller if the population of [^15^N]-FDX1-FNR complexes became smaller upon addition of FDX2. If the affinities of FNR to FDX1 and FDX2 were the same, addition of equal amounts of [^15^N]-FDX1 and non-labelled FDX2 to FNR would halve the chemical shift changes of [^15^N]-FDX1 caused by complex formation with FNR compared to those without non-labelled FDX2. In our experiment, we mixed [^15^N]-FDX1, FDX2 and FNR at a molar ratio of 1:3:3; i.e. the amount of FDX2 was three times the amount of [^15^N]-FDX1. In such a condition, observing a Δ*δ*_with FDX2_/Δ*δ*_without FDX2_ ratio larger than 0.5 would already be enough to assume that FDX1 prevents FDX2 from interacting with FNR. However, to be safe we set the threshold to 0.66 (=2/3) and assumed that a Δ*δ*_with FDX2_/Δ*δ*_without FDX2_ ratio larger than 0.66 shows that [^15^N]-FDX1 competitively inhibits FDX2 from interacting with FNR.

### Absorption spectroscopy

Samples were adjusted to a chlorophyll concentration of 20 μg mL^−1^ in 20 mM HEPES–KOH pH 7.2 containing 10% (w/v) Ficoll and dark incubated for at least 10 min prior to the measurements. Absorption spectra were recorded by means of a pump and probe LED-based Joliot-type dual beam spectrophotometer. For carotenoid electrochromic bandshift (ECS) measurements, the setup included a white detection LED, a 520 and a 546 nm interference filter (10 nm bandwidth) and BG39 bandpass filters in front of the photodiodes as well as a dye laser pumped by a frequency-doubled Nd:Yag laser. To correct for unspecific contributions, absorption changes measured at 546 nm were subtracted to those measured at 520 nm. To assess photochemical charge separation events at the two photosystems, the amplitude of the initial rise in response to a saturating single turnover flash was evaluated. The ECS signal produced by PSI alone was discriminated via addition of PSII inhibitors (1 mM HA, 20 µM DCMU), while contributions of PSII were calculated as the fraction being sensitive to these inhibitors. This procedure allows a precise quantification of the stoichiometry of functional reaction centres (PSI/PSII). For P700/P700+ redox change measurements the setup included a detection LED peaking at 700 nm, a 705 and a 740 nm interference filter (10 nm bandwidth) and two RG695 long-pass cut-off filters in front of the photodiodes. To correct for unspecific contributions, absorption changes measured at 740 nm were subtracted to those measured at 705 nm. The amount of photooxidizable P700 (i.e. active PSI reaction centres) was estimated as the maximum amplitude induced by a saturating pulse (30 ms) following 10 s of continuous illumination (160 μE m^−2^ s^−1^; 630 nm) in the presence of 20 µM DCMU.

## Results

### In vitro interaction of soluble and PSI-bound FNR with FDX1 and FDX2

**Fig. 1:**
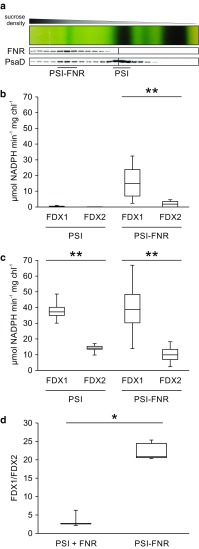
Light-dependent NADPH production mediated by PSI complexes and PSI–FNR complexes via FDX1 versus FDX2. **a** Complexes were isolated from anaerobic thylakoids of *C. reinhardtii* wild-type strain cc124 by SDG centrifugation upon solubilization with β-DDM. Samples for immunoblots were adjusted to equal volume (100 µL). **b, c** Light-dependent NADPH production rates in the absence (**b**) and presence (**c**) of additional soluble FNR (0.5 µM). 3 ≤ *n* ≤ 15. Whiskers: Min to Max. Statistically significant differences are indicated by *asterisks* (** ≙ *p* < 0.001). Samples contained isolated PSI particles (5 μg mL^−1^ chl), 20 mM Tricine–KOH (pH 7.4), 3 mM MgCl_2_, 2 mM ascorbate, 60 µM DCPIP, 40 µM PC, 4 µM FDX1/2 and 0.5 mM NADP^+^. To assess light-dependent NADPH production, absorption at 340 nm was measured prior to and after 15 and 30 s of illumination. Rates of PSI–FNR fractions were normalized to the PSI level of PSI fractions. **d** Relative NADPH production rates (FDX1/FDX2) mediated by PSI particles in the presence of soluble FNR and by PSI–FNR particles. One asterisk indicates a statistically significant difference (*p* = 0.008)

To elucidate whether FNR is potentially capable of differentially modulating electron flow downstream of PSI, we investigated its substrate specificity using the example of PSI-dependent NADP^+^ reduction via FDX1 versus FDX2. For this purpose, PSI particles were isolated by SDG ultracentrifugation of β-DDM solubilized thylakoid membranes extracted from anaerobic *C. reinhardtii* wild-type cells (Fig. 1a). Two SDG fractions including two distinct types of PSI complexes were utilized for NADP^+^ photoreduction assays: PSI–LHCI (referred to as PSI) and the high molecular weight complex (i.e. the putative CEF supercomplex) comprising not only PSI–LHCI–LHCII and FNR, but also Cyt b_6_f, PGRL1, PETO, ANR1 and CAS (referred to as PSI–FNR). Virtually no NADPH production was observed in PSI fractions, while complex-associated FNR in PSI–FNR fractions mediated NADP^+^ photoreduction via both FDX1 and FDX2. Notably, NADPH production rates obtained with FDX1 were significantly higher than with FDX2 (*p* < 0.001; Fig. 1b). Addition of soluble FNR generally enhanced NADP^+^ photoreduction and resulted in equal NADPH production rates in PSI and PSI–FNR fractions in assays performed with FDX1, whereas assays utilizing FDX2 yielded slightly higher NADPH production rates in PSI compared to PSI–FNR fractions. Again, NADPH production rates obtained with FDX1 were significantly higher than with FDX2, indicating a higher electron transfer efficiency from FDX1 to FNR compared to FDX2 (*p* < 0.001; Fig. 1c). However, while in PSI fractions FDX1 was a 2.6-fold more efficient electron donor to soluble FNR than FDX2, the superior activity of FNR with FDX1 compared to FDX2 was enhanced significantly to a factor of 23 in PSI–FNR fractions (*p* = 0.008; Fig. 1d). This observation suggests that relative to FDX1, electron transfer from FDX2 to soluble FNR is more efficient than to complex-associated FNR.

**Fig. 2:**
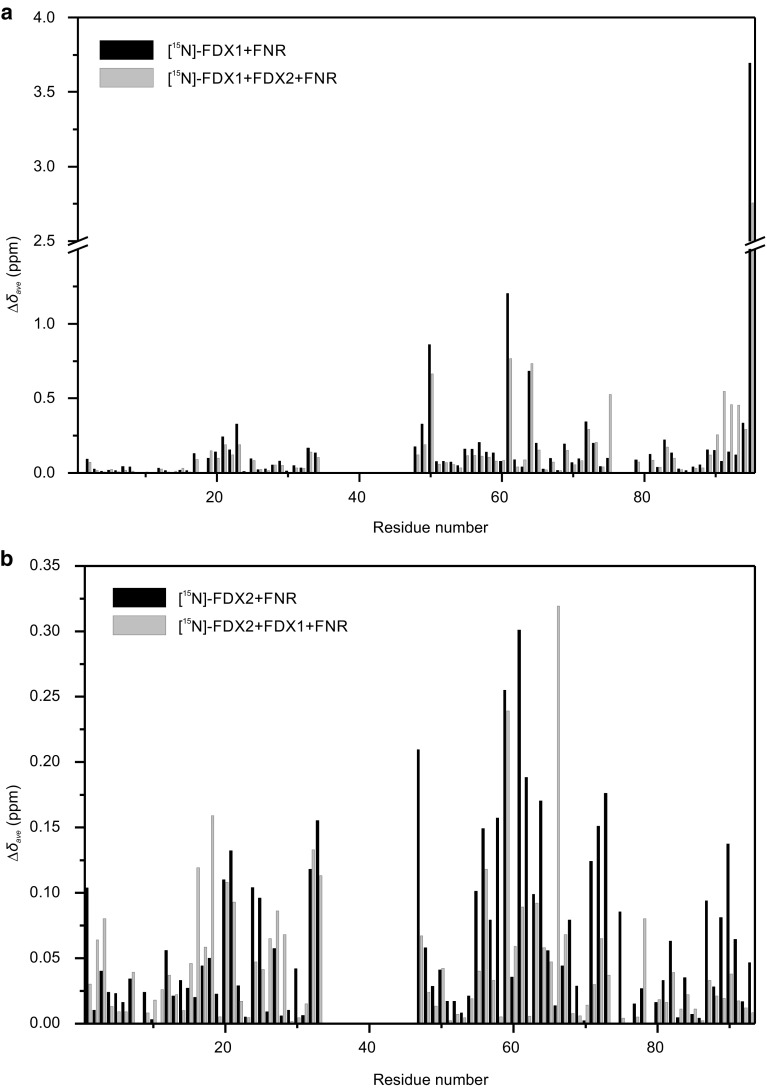
Comparison of chemical shift perturbations of [^15^N]-FDX1 and [^15^N]-FDX2. **a** Chemical shift changes of [^15^N]-FDX1 upon complex formation with FNR in the presence of FNR (*black*) and in the presence of both FNR and FDX2 (*grey*). **b** Chemical shift changes of [^15^N]-FDX2 upon complex formation with FNR in the presence of FNR (*black*) and in the presence of both FNR and FDX1 (*grey*). The scales of the *vertical axes* in the two graphs are different. The ^1^H and ^15^N chemical shifts are merged for each amide group according to the equation described in “Materials and Methods”

To further investigate whether the decreased electron transfer efficiency from FDX2 to FNR is partially due to FNR discriminating between FDX1 and FDX2, we set up a competition experiment based on NMR 2D ^1^H–^15^N HSQC spectra of [^15^N]-labelled FDXs. Under a fast exchange regime on the NMR time scale, the magnitude of chemical shift perturbations due to complex formation is proportional to the molar ratio of the complex to free molecules. If FNR was not able to distinguish between the two FDXs and had an equal affinity for both, changes in the chemical shifts of [^15^N]-FDX1 would decrease upon addition of a large amount of non-labelled FDX2. In contrast, if FNR was able to distinguish between the two FDXs with a higher affinity for FDX1, the chemical shift values of [^15^N]-FDX1 would show minimal change upon addition of non-labelled FDX2 (or vice versa). Initially, chemical shift perturbations of [^15^N]-FDX1 were assessed in the presence of FNR, but without FDX2 (Fig. 2a, black bars). In comparison, even in the presence of three times more FDX2 than [^15^N]-FDX1, chemical shift changes of [^15^N]-FDX1 were similar to those observed in the absence of FDX2 (Fig. 2a, grey bars). Since some residues could not be detected because of broadening by the nearby paramagnetic irons of FDX, 75 perturbed residues of [^15^N]-FDX1 were observed in total. Among those, 56 residues (75% of the total) exhibited a Δ*δ*_with FDX2_/Δ*δ*_without FDX2_ larger than 0.66, indicating that FNR preferentially interacts with FDX1 rather than FDX2. Next, the reciprocal experiment was conducted by replacing [^15^N]-FDX1 with [^15^N]-FDX2. Chemical shift perturbations in 76 residues of [^15^N]-FDX2 were detected in the presence of FNR (Fig. 2b, black bars), but their magnitudes were much smaller than those observed for [^15^N]-FDX1. (Note that the scale of the vertical axis in Fig. 2b is magnified compared to Fig. 2a). In the presence of FDX1, only 36 residues of [^15^N]-FDX2 (corresponding to 47% of the total) featured a Δ*δ*_with FDX1_/Δ*δ*_without FDX1_ ratio larger than 0.66, while the remaining 30 residues did not show significant Δ*δ*_with FDX1_/Δ*δ*_without FDX1_ ratios (Fig. 2b, grey bars). These results confirm that even in solution, FNR distinguishes between FDX1 and FDX2, with preferential binding to FDX1.

### FNR activity/localization and high-light response of PGR5/PGRL1 knockout mutants

**Fig. 3:**
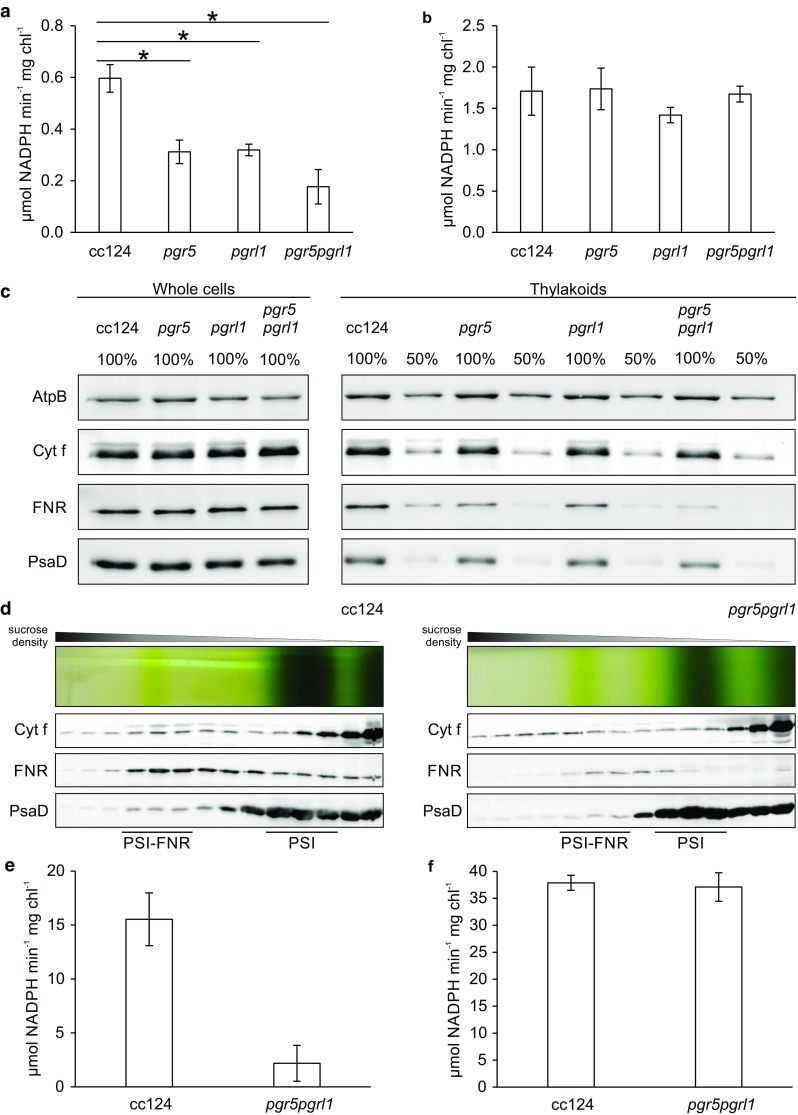
**a**–**c** Light-dependent NADPH production mediated by thylakoid membranes isolated from anaerobic wild-type (cc124), *pgr5, pgrl1* and *pgr5pgrl1* cells and protein abundances in anaerobic whole cells and isolated thylakoids. **a, b** Light-dependent NADPH production rates in the absence (**a**) and presence (**b**) of additional soluble FNR (0.5 µM). Values represent means (SEM) of 3 ≤ *n* ≤ 7 biological replicates. One asterisk indicates a statistically significant difference (*p* = 0.002). Samples contained isolated thylakoids (66 μg chl mL^−1^), 50 mM HEPES–KOH (pH 7.5), 10 mM MgCl_2_, 0.1 μM nigericin, 0.05% β-DDM, 2 mM ascorbate, 60 μM DCPIP, 5 μM PC, 10 μM FDX1 and 0.5 mM NADP^+^. To assess light-dependent NADPH production, absorption was measured prior to and after 20 and 40 s of illumination (600 μE m^−2^ s^−1^). **c** Cyt f, FNR and PsaD abundance. Samples for immunoblots were adjusted to equal chlorophyll content (2 µg). AtpB was used as a loading control. **d**–**f** Light-dependent NADPH production mediated by PSI complexes and PSI–FNR complexes isolated from *C. reinhardtii* wild-type strain cc124 in comparison to *pgr5pgrl1*. **d** Complexes were isolated from anaerobic thylakoids of cc124 and *pgr5pgrl1* by SDG centrifugation upon solubilization with β-DDM. Samples for immunoblots were adjusted to equal volume (100 µL). **e, f** Light-dependent NADPH production rates mediated by PSI–FNR complexes in the absence (**e**) and PSI complexes in the presence (**f**) of additional soluble FNR (0.5 µM). Values represent means (SEM) of 2 ≤ *n* ≤ 15 biological replicates. Samples contained isolated PSI particles (5 μg mL^−1^ chl), 20 mM Tricine–KOH (pH 7.4), 3 mM MgCl_2_, 2 mM ascorbate, 60 µM DCPIP, 40 µM PC, 4 µM FDX1 and 0.5 mM NADP^+^. To assess light-dependent NADPH production, absorption at 340 nm was measured prior to and after 15 and 30 s of illumination. Rates of PSI–FNR fractions were normalized to the PSI level of PSI fractions

To explore the potential role of FNR in the regulation of photosynthetic electron transport, we expanded the in vitro NADP^+^ photoreduction assays to a *C. reinhardtii* PGR5, a PGRL1 and a double knockout mutant. Notably, thylakoids isolated from anaerobic cultures of these mutants featured significantly lower NADPH production rates than the wild-type (*p* = 0.002; Fig. 3a). However, addition of soluble FNR generally enhanced NADP^+^ photoreduction and restored wild-type NADPH production rates (Fig. 3b). This finding suggests a diminished amount of membrane-associated FNR in the absence of PGRL1 and/or PGR5. Immunoblots confirmed a decreased FNR abundance in *pgr5, pgrl1* and *pgr5pgrl1* on thylakoid level, while FNR expression on whole cell level was comparable. Similarly, the amounts of PsaD and Cyt f were unaltered (Fig. 3c). SDG ultracentrifugation of β-DDM solubilized thylakoid membranes isolated from anaerobic *pgr5pgrl1* cells did still yield two types of PSI complexes as in the wild-type cc124. However, the amount of FNR and PSI retained in the high molecular weight fractions was diminished and the distribution of Cyt b_6_f was shifted relative to FNR and PSI (Fig. 3d), implicating that the stability of the whole complex may be impaired in the absence of PGR5 and PGRL1. In line with this observation, PSI–FNR samples derived from *pgr5pgrl1* mediated lower NADPH production rates compared to cc124 (Fig. 3e), while overall PSI activity in the presence of soluble FNR was comparable between mutant and wild-type (Fig. 3f).

**Fig. 4:**
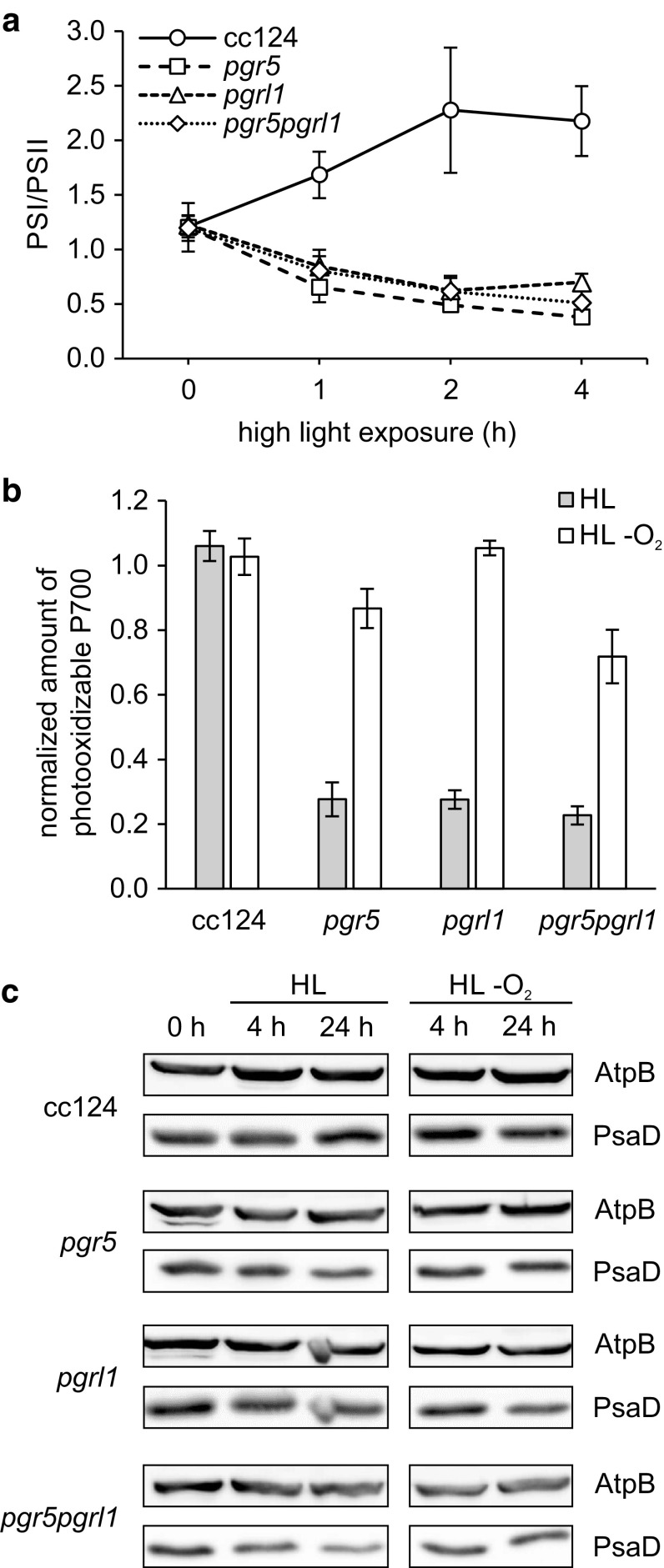
High-light response of the *C. reinhardtii* wild-type strain cc124 in comparison to *pgr5, pgrl1* and *pgr5pgrl1*. **a** Functional reaction centre stoichiometry versus duration of the high-light exposure. Data points represent means (SEM) of *n* = 3 biological replicates. PSI/PSII was determined by the initial rise of the ECS signal at 520 nm upon a saturating single turnover flash in the absence and presence of PSII inhibitors (1 mM HA, 20 µM DCMU). Cells were shifted to photoautotrophic conditions (4 µg chl mL^−1^; HSM) prior to the HL exposure (200 µE m^−2^ s^−1^). **b** Amount of photooxidizable P700 after 4 h high light (HL) in the presence and absence of O_2_ (normalized to the respective amount at 0 h). Data points represent means (SEM) of *n* = 3 biological replicates. The amount of photooxidizable P700 was assessed via the maximum absorption change at 705 nm in response to 10 s continuous illumination (160 µE m^−2^ s^−1^, 340 nm) followed by a saturating pulse (30 ms) in the presence of 20 µM DCMU. Cells were bubbled with argon (4 µg chl mL^−1^; TAP) to induce anoxic conditions during the high-light exposure (200 µE m^−2^ s^−1^). Samples for absorption spectroscopy contained 20 µg chl mL^−1^ in 20 mM HEPES–KOH (pH 7.2) 10% w/v Ficoll. **c** PsaD abundance prior to and 4 h and 24 h after a shift to high light in the presence and absence of O_2_. Samples for immunoblots were adjusted to equal chlorophyll content (2.5 µg). AtpB was used as a loading control

To further investigate potential functions of PGR5 and PGRL1, we examined the response of the same mutants to high-light stress. Both high light and anoxia cause the accumulation of reducing equivalents in the chloroplast stroma and induce the formation of the putative CEF supercomplex, which recruits FNR to the membrane (Takahashi et al. 2013; Bergner et al. 2015). In *pgr5, pgrl1* and *pgr5pgrl1*, PSI was the primary target of photodamage, as evidenced by a decline of the functional reaction centre stoichiometry over time in high light. In contrast, PSI/PSII gradually augmented in the wild-type during the first 4 h in high light, implying predominant photoinhibition of PSII (Fig. 4a). The observed inactivation of PSI in the mutants in response to short-term high-light stress entailed PSI degradation on a longer time scale, which was detected via immunoblots monitoring PsaD levels (Fig. 4c). This indicates the absence of a regulatory feedback in *pgr5, pgrl1* and *pgr5pgr11*, which protects PSI at the expense of PSII in the wild-type. Notably, elimination of O_2_ during the high-light exposure partially preserved PSI both on a functional and structural level (Fig. 4b, c). This observation points towards enhanced electron donation to O_2_ and ROS-mediated PSI photodamage in *pgr5, pgrl1* and *pgr5pgr11*, presumably arising from deregulated photosynthetic electron transport and subsequent PSI acceptor side limitation, but potentially also related to low P700 oxidation efficiency due to diminished FNR binding to PSI.

## Discussion

Herein, we investigated the functional importance of the association of FNR with the photosynthetic apparatus in *C. reinhardtii*. Our data indicate that binding of FNR to a PSI supercomplex potentially modulates the interaction with FDX1 and FDX2, reinforcing the preference for FDX1 over FDX2. The association of FNR with the thylakoid membrane including PSI is impaired in the absence of PGR5 and/or PGRL1. This finding implies that both proteins, directly or indirectly, contribute to the recruitment of FNR to the thylakoid membrane, which may form part of a photoprotective mechanism to oxidize PSI.

### Soluble FNR interacts differentially with FDX1 and FDX2

Although the primary sequence similarity between FDX1 and FDX2 is high (82%), structural modelling predicted differences in the surface charge distribution. Likewise, the midpoint potential of FDX2 (−321 mV/−331 mV) is more positive than the midpoint potential of FDX1 (−398 mV), reflecting the respective clustering with root-type and leaf-type FDXs (Terauchi et al. 2009; Boehm et al. 2015). These distinct properties impact the interaction and electron transfer between FNR and FDX1 or FDX2. In this study, we observed significantly higher NADPH production rates mediated by FNR via FDX1 compared to FDX2 (Fig. 1b, c). In line with previous reports (Peden et al. 2013), this finding indicates that FDX1 is a more efficient electron donor to FNR than FDX2. The observed difference in maximum electron transfer capacity may be attributed to differences in both electron transfer efficiency and affinity. In line with this, analysis of chemical shift perturbations (Fig. 2a, b) demonstrated that FDX1 competitively inhibits FDX2 interaction with FNR, implying a higher affinity of FNR to FDX1 compared to FDX2. This finding is in contrast to other previous work, where lower K_M_ values were reported for the interaction of FNR with FDX2 compared to FDX1 (Terauchi et al. 2009; Boehm et al. 2015). Relating to the data generated by Terauchi et al., this apparent contradiction is resolved easily, as these authors measured FNR activity in the heterotrophic direction (i.e. the interaction of reduced FNR with FDX), which has a different set of catalytic constants. In terms of physiology, a lower K_M_ for the interaction of reduced FNR with FDX2 compared to FDX1 is not surprising, as FDX2 is predicted to be a root-type FDX. This altered relative affinity depending on the redox state of the enzyme and substrate is in line with the presumed function of *C. reinhardtii* FNR in conducting both NADP^+^ photoreduction (higher affinity for reduced FDX1) and NADPH-dependent FDX reduction to support heterotrophic metabolism (higher affinity for oxidized FDX2). Furthermore, we found that even when measuring FNR activity in the photosynthetic direction, the electron transfer efficiency with FDX2 was significantly higher when FNR was soluble (Fig. 1d)—the expected localisation of heterotrophic FNR (Korn et al. 2009). Still, we cannot account for the differences from Boehm et al., except to note that the same group previously showed similar results to us, with a higher electron transfer efficiency for FDX1 than FDX2 in the photosynthetic direction (Peden et al. 2013).

### Association of FNR with a PSI supercomplex modulates interaction with FDX1 and FDX2

**Fig. 5:**
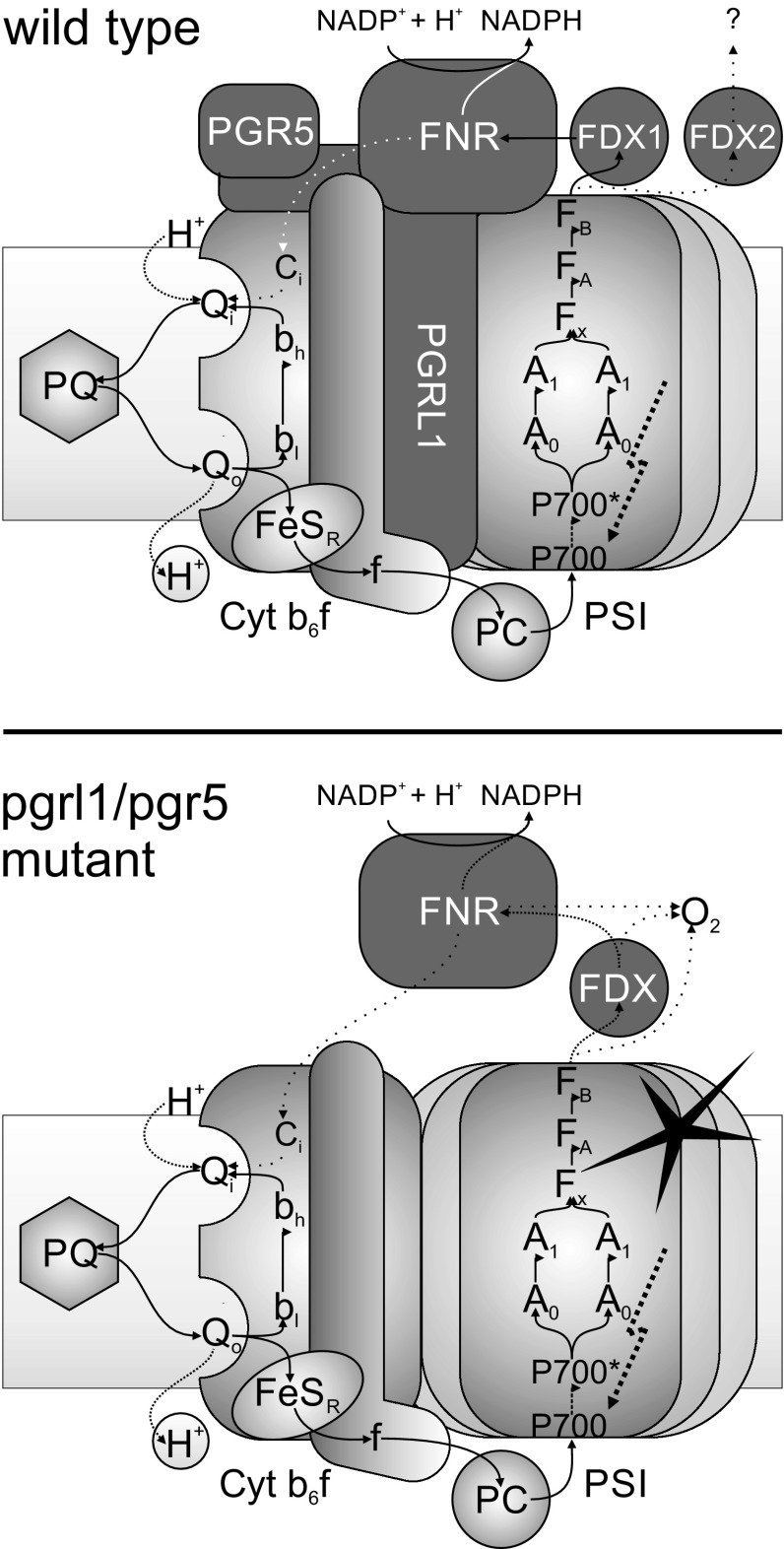
Schematic model illustrating the potential relationships established in this work between different components of the photosynthetic apparatus in *C. reinhardtii*. In conditions where FNR is membrane bound, FDX2 potentially channels electrons derived from PSI towards other processes than NADPH production. PGR5 and PGRL1 contribute to the recruitment of FNR to PSI and/or Cyt b_6_f. In the absence of PGR5 and/or PGRL1, impaired membrane association of FNR results in PSI acceptor side limitation and enhanced electron donation to O_2_

Comparing NADPH reduction rates mediated by soluble FNR versus complex-associated FNR (Fig. 1d) revealed that the binding of FNR to PSI impacts interaction with FDX1 and FDX2, presumably through structural rearrangement further enhancing the preference for FDX1 over FDX2. This finding is in line with a role of complex-associated FNR in NADP^+^ photoreduction (Korn et al. 2009), as well as with previous studies reporting that spinach FNR becomes more efficient at NADP^+^ photoreduction upon binding to thylakoid membrane complexes (Forti et al. 1983; Forti and Bracale 1984). As FDX2 preferentially interacts with soluble FNR, it might be involved in channelling electrons derived from PSI towards other processes than NADPH production in conditions when FNR is membrane-bound (Fig. 5). At this point, the physiological role of *C. reinhardtii* FDX2 is elusive. However, the in vitro assays presented here demonstrate the high substrate specificity of *C. reinhardtii* FNR and support the idea of photosynthetic electron transport partitioning via differential association of FNR with thylakoid membrane complexes, thereby channelling electrons towards distinct routes.

### PGR5 and PGRL1 impact the recruitment of FNR to the thylakoid membrane

Notably, the membrane association of FNR is impaired in the absence of PGR5 and/or PGRL1 (Fig. 3). At this point, it is impossible to differentiate whether this phenomenon merely represents another symptom of the multifaceted phenotype of these mutants—or if it is directly related to the absence of PGR5 and/or PGRL1. The question remains how might PGR5 and/or PGRL1 mechanistically impact the association of FNR with the thylakoid membrane? PGR5 (135 amino acids) is associated with the stromal side of the membrane (Munekage et al. 2002). PGRL1 (324 amino acids) possesses two transmembrane domains (DalCorso et al. 2008), performs iron-dependent redox-induced conformational changes and features six redox-active cysteine residues, four of which form a rubredoxin fold presumably facilitating the binding of an iron-containing cofactor (Petroutsos et al. 2009; Hertle et al. 2013). PGR5 accumulation requires the presence of PGRL1, whereas PGR5 promotes PGRL1 stability (Petroutsos et al. 2009; Johnson et al. 2014). This mutual dependence also complicates the specific attribution of phenotypes to the absence of either of the two proteins. PGRL1 forms homodimers being destabilized by TRX. The negatively charged N-terminal loop of PGRL1 likely interacts with the positively charged regions of PGR5, resulting in the formation of a heterodimer (DalCorso et al. 2008). Copurification indicated the PGR5–PGRL1 complex associates with PSI. Moreover, split-ubiquitin and yeast two-hybrid assays revealed that PGRL1 interacts with FDX and FNR. In addition, both PGRL1 and PGR5 interact with Cyt b_6_f, as confirmed by co-immuno-precipitation assays (DalCorso et al. 2008; Hertle et al. 2013). Therefore, altered binding of FNR to PSI or Cyt b_6_f as a direct effect of the absence of PGRL1 and/or PGR5 is plausible. Notably, in *A. thaliana fnr1* × *fnr2* plants containing 53% of wild-type FNR levels, PGRL1 expression was induced by 174% (Lintala et al. 2012), possibly to reinforce binding of residual FNR to the thylakoid membrane. Based on their observation that FNR, PGRL1, PETO and ANR1 comigrated in SDGs of solubilized *C. reinhardtii* thylakoids isolated from oxic conditions, Takahashi and coworkers proposed that these proteins might coreside in membrane microdomains prior to the association with Cyt b_6_f and PSI upon a shift to reducing conditions (Takahashi et al. 2013, 2016). The TRX-dependent destabilization of PGRL1 homodimers and the subsequent PGRL1–PGR5 heterodimerzation (Hertle et al. 2013) are a potential mechanism to induce the formation of the putative CEF supercomplex in such a redox-dependent way. ANR1 potentially senses the PQ pool redox poise or the electrochemical proton gradient and may thereby initiate a remodelling of this membrane microdomain facilitating the interaction of FNR with Cyt b_6_f (Takahashi et al. 2016), potentially via PGR5 and/or PGRL1. Indeed, interaction between ANR1 and PGRL1 has been confirmed by BIFC analyses (Terashima et al. 2012). Notably, CEF supercomplex formation was observed in both ANR1 knockdown mutants (Terashima et al. 2012) and in *pgr5pgrl1* (Fig. 3d). Likewise, the diminished in vitro NADP^+^ photoreduction rates observed in thylakoids isolated from *pgr5, pgrl1* and *pgr5pgrl1* (Fig. 3) presumably represent an FNR dose effect, with the catalytic activity of membrane-associated FNR probably unaltered by the absence of PGR5 and/or PGRL1. Overall, these observations suggest that PGRL1 and PGR5 might play a structural/regulatory role in FNR membrane recruitment in *C. reinhardtii* (Fig. 5). To decide whether this conclusion is applicable to vascular plants lies beyond the scope of this article, but mechanistic differences between green algae and land plants in terms of FNR membrane binding are plausible, as orthologues of the tethering proteins in vascular plants are absent in the unicellular alga. Furthermore, based on in vitro experiments on vascular plants, PGLR1 has been attributed a direct role as FQR in antimycin A-sensitive CEF (Hertle et al. 2013), so that functional differences between PGRL1 from green algae (where CEF is not inhibited by antimycin A) and land plants are likely. Although the PSI–Cyt b_6_f complex recently isolated from *A. thaliana* lacks FNR and PGRL1 (Yadav et al. 2017), binding of FNR to PSI has been described in barley (Andersen et al. 1992) and association of FNR with Cyt b_6_f has been reported in spinach (Clark et al. 1984; Zhang et al. 2001). As already mentioned, the *A. thaliana* PGR5–PGRL1 complex associates with PSI, both PGRL1 and PGR5 interact with Cyt b_6_f and PGRL1 interacts with FNR (DalCorso et al. 2008; Hertle et al. 2013). Only further analyses addressing the molecular role of PGR5 and PGRL1 as well as the physiological role of FNR membrane binding in both green algae and land plants may elucidate whether parts of the underlying mechanisms are evolutionary conserved.

### Potential roles of PGR5, PGRL1 and FNR in photosynthetic electron transport regulation and PSI photoprotection

The absence of PGR5 and/or PGRL1 results in a severe deregulation of electron transfer in response to high light, with PSI being the primary target of photodamage in *pgr5, pgrl1* and *pgrl1pgr5*, while PSII is predominantly affected in the wild-type (Fig. 4). A similar phenomenon was observed in *C. reinhardtii pgrl1* after a shift from high to low CO_2_ conditions (Dang et al. 2014) as well as in *A. thaliana pgr5* following a shift from growth to high light (Tikkanen et al. 2014). In line with this, overexpression of PGR5 in *A. thaliana* resulted in enhanced PSI stability but reduced PSII stability in response to high light (Long et al. 2008). Hence, PGR5 and PGRL1 appear crucial in establishing PSII as the “predetermined breaking point” of the photosynthetic electron transport chain. In situations where excitation flux exceeds metabolic capacity, *pgr5, pgrl1* and *pgr5pgrl1* fail to shift the rate-limiting step of LEF from the PSI acceptor side to Cyt b_6_f due to impaired luminal acidification, in line with conclusions related to *A. thaliana pgr5* in response to fluctuating light (Suorsa et al. 2012). Notably, Joliot and Johnson observed in *A. thaliana* that the addition of low concentrations of the uncoupler nigericin mimicked the *pgr5* phenotype: The induction of qE-dependent NPQ and more importantly the extent of photosynthetic control was lowered, resulting in predominant reduction of P700^+^ in the light and subsequent PSI photoinhibition (Joliot and Johnson 2011). In the PGR5 and/or PGRL1 knockout mutants, the impaired luminal acidification is a consequence of decreased proton influx due to less efficient FQR-dependent CEF and/or increased proton efflux due to alterations of the ATPase conductivity (Avenson et al. 2005; Wang et al. 2014).

On a short time scale, perturbation of the regulatory feedback loop linking metabolic capacity and excitation flux results in over-reduction of stromal electron acceptors in the mutants. On a long time scale, PSI acceptor side limitation entails the inactivation and degradation of PSI reaction centres due to photooxidative damage (Fig. 4 a, c). A similar effect has been described previously in *A. thaliana pgr5* as well as in *C. reinhardtii pgr5* and *pgrl1* (Suorsa et al. 2012; Johnson et al. 2014; Kukuczka et al. 2014). The observed preservation of PSI in the absence of O_2_ (Fig. 4 b, c) supports the idea that enhanced electron donation to O_2_ results in ROS-mediated PSI photodamage in *pgr5, pgrl1* and *pgr5pgrl1* (Fig. 5). In agreement with this hypothesis, increased H_2_O_2_ production has been reported in *C. reinhardtii pgrl1* exposed to high light (Dang et al. 2014). Likewise, ROS scavenging enzymes are upregulated in *A. thaliana pgr5* seedlings exposed to fluctuating light to potentially protect PSI from enhanced ROS production (Suorsa et al. 2012), and FLVA and B levels are elevated in *C. reinhardtii pgrl1* to mediate “controlled” O_2_ photoreduction in response to high light and also following a shift from high to low CO_2_ (Dang et al. 2014). In line with this finding, overexpression of *Physcomitrella patens* FLVA and FLVB abolishes PSI acceptor side limitation in *A. thaliana pgr5* plants (Yamamoto et al. 2016).

The data presented here indicate that a contributory factor to PSI photodamage in the absence of PGR5 and/or PGRL1 (Fig. 4) might be diminished binding of FNR to PSI complexes (Fig. 3). As thylakoid membrane association of the enzyme potentially influences its activity and affinity (Fig. 1d), loss of FNR from these supercomplexes probably compounds PSI acceptor side limitation. A sustained oxidation of P700 in the light is essential to prevent PSI photoinhibition (Shimakawa et al. 2016; Takagi et al. 2017) and the recruitment of FNR to PSI may provide a high P700 oxidation efficiency. Binding of FNR to PSI might also affect the efficiency of electron transfer between FDX1 and FNR, since significantly more H_2_ is produced in the absence of PGR5 and/or PGRL1 under anoxic conditions (Tolleter et al. 2011; Godaux et al. 2015; Steinbeck et al. 2015; Chen et al. 2016). Assuming that electron transfer between FNR and FDX1 is compromised due to altered FNR membrane recruitment, more reduced FDX1 would be available for HydA reduction, resulting in enhanced H_2_ production in the mutants. Notably, FNR was indeed shown to compete with HydA for reduced FDX in vitro (Yacoby et al. 2011). Furthermore, the binding of FNR to PSI has been proposed to play a direct photoprotective role: The reduction of NADP^+^ requires two electrons, while FDX transfers first one electron to the oxidized FAD cofactor and upon rebinding a second electron to the semiquinone radical, finally resulting in the hydroquinone. Recruitment of FNR to the membrane might therefore enhance the efficiency of electron transfer from PSI to NADP^+^ during LEF, decreasing the lifetime of the hazardous semiquinone radical and preventing deleterious electron donation to O_2_ (Takahashi et al. 2014; Bergner et al. 2015). Likewise, FNR abundance and location in general have been shown to impact ROS production (Kozuleva et al. 2016). Last but not least, the association of FNR with Cyt b_6_f has been proposed to promote CEF, establishing FNR relocation in response to the stromal redox poise as central hub of photosynthetic electron transfer partitioning (Joliot and Johnson 2011; Goss and Hanke 2014). Therefore, disturbed association of FNR with the thylakoid membrane may partly explain PGR5/PGRL1 knockout-related phenotypes. Intriguingly, a tobacco FNR knockdown mutant exhibited a similar phenotype, i.e. deficient qE-dependent NPQ induction, reduced CEF efficiency and higher susceptibility to PSI photoinhibiton (Hald et al. 2008; Joliot and Johnson 2011). In conclusion, PGR5 and PGRL1 play a major, but mechanistically elusive role in the regulation of photosynthetic electron transport and PSI photoprotection in *C. reinhardtii*. Our data suggest that diminished amounts of membrane-associated FNR in the absence of PGR5 and/or PGRL1 potentially alter electron flow in vivo and thereby contribute to the multifaceted phenotype of the corresponding knockout mutants.

## Figures and Tables

**Fig. 6:**
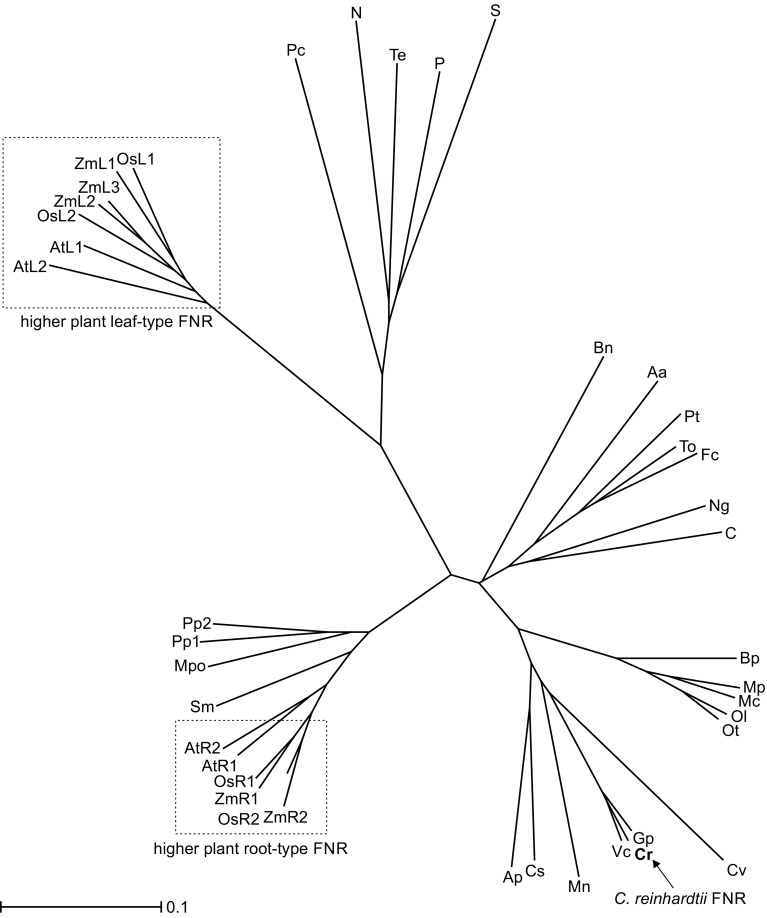
Phylogenetic tree showing the diversity of FNR sequences in photosynthetic organisms. Mature protein sequences were aligned with Clustal Omega (http://www.ebi.ac.uk/Tools/%20msa/clustalo) and the phylogenetic tree was estimated based on neighbour joining implemented by splitstree (D.H. Huson and D. Bryant, Application of Phylogenetic Networks in Evolutionary Studies, Molecular Biology and Evolution, 23(2):254–267, 2006; software available from http://www.splitstree.org). Vascular plant leaf and root FNR sequences are referred to as L and R, FNR sequence assignments are as follows (in alphabetical order): Aa (*Aureococcus anophagefferens*): XP_009035092.1; Ap (*Auxenochlorella protothecoides*): XP_011400423.1; At (*Arabidopsis thaliana*) L1: NP_201420.1, L2: NP_001077566.1, R1: NP_001190682.1, R2: NP_849734.1; Bn (*Bigelowiella natans*): AAP79145.1; Bp (*Bathycoccus prasinos*): XP_007510909.1; C (*Chrysochromulina* sp.): KOO53489.1; Cr (*C. reinhardtii*): XP_001697352.1; Cs *(Coccomyxa subellipsoidea*): XP_005644145.1; Cv (*Chlorella variabilis*): XP_005848997.1; Fc (*Fragilariopsis cylindrus*): OEU17081.1; Gp (*Gonium pectorale*): KXZ46591.1; Mc (*Micromonas commoda*): XP_002500720.1; Mn (*Monoraphidium neglectum*): XP_013905631.1; Mp (*Micromonas pusilla*): XP_003064407.1; Mpo (*Marchantia polymorpha*): OAE27096.1; N (*Nostoc* sp.): WP_010998260.1; Ng (*Nannochloropsis gaditana*): XP_005853875.1; Ol (*Ostreococcus lucimarinus*): XP_001422240.1; Os (*Oryza sativa*) L1: BAS95764.1, L2: BAS76543.1, R1: BAF13390.1, R2: BAF20804.1; Ot (*Ostreococcus tauri*): XP_003084170.1; P (*Pseudanabaena* sp.): WP_015165197.1; Pc (*Paulinella chromatophora*): YP_002049405.1; Pp (*Physcomitrella patens)* 1: XP_001770917.1, 2: XP_001774270.1; Pt (*Phaeodactylum tricornutum*): XP_002180511.1; S (*Synechocystis* sp.): WP_010873079.1; Sm (*Selaginella moellendorffii*): XP_002982108.1; Te (*Thermosynechococcus elongatus*): WP_011057053.1; To (*Thalassiosira oceanica*): EJK72001.1; Vc (*Volvox carteri*): XP_002954986.1; Zm (*Zea mays*) L1: BAA88236.1, L2: BAA88237.1, L3: ACF85815.1, R1: ACG39703.1, R2: ACG35047.1

## Appendix

See Fig. 6.

## Abbreviations

ANR1: Anaerobic response 1
BiFC: Bimolecular fluorescence complementation
CAS: Calcium sensor
CEF: Cyclic electron flow
Cyt b_6_f: Cytochrome b_6_f complex
DCMU: 3-(3,4-Dichlorophenyl)-1,1-dimethylurea
DCPIP: Dichlorophenolindophenol
DDM: Dodecyl-d-maltoside
FDX: Ferredoxin
FNR: FDX:NADP^+^ oxidoreductase
FQR: FDX:quinone oxidoreductase
HA: Hydroxylamine
HSQC: Heteronuclear single-quantum coherence
HydA: Hydrogenase
LEF: Linear electron flow
LHC: Light harvesting complex
LIR1: Light-induced rice 1
NPQ: Non-photochemical Quenching
PC: Plastocyanin
PFR: Pyruvate:FDX oxidoreductase
PGR5: Proton Gradient Regulation 5
PGRL1: PGR5-like photosynthetic phenotype 1
PQ: Plastoquinone
PSI: Photosystem I
TROL: Thylakoid rhodanese-like protein
TRX: Thioredoxin
